# Particulate matter increases *Cutibacterium acnes*-induced inflammation in human epidermal keratinocytes via the TLR4/NF-κB pathway

**DOI:** 10.1371/journal.pone.0268595

**Published:** 2022-08-10

**Authors:** Hyun Ha Noh, Sun Hye Shin, Yoon Jin Roh, Nam Ju Moon, Seong Jun Seo, Kui Young Park

**Affiliations:** 1 Department of Dermatology, Chung-Ang University College of Medicine, Seoul, Korea; 2 Department of Ophthalmology, Chung-Ang University Hospital, Seoul, Korea; National Center for Toxicological Research, UNITED STATES

## Abstract

Recent studies have demonstrated that particulate matter (PM) can induce oxidative stress and inflammatory responses that are related to the development or exacerbation of several inflammatory dermatoses. However, the effect of PM on acne vulgaris has yet to be determined. In this study, we induced acne-like inflammation in HEKn cells with several concentrations of *Cutibacterium acnes (C*. *acnes)* and *Staphylococcus aureus* peptidoglycan (PGN) to investigate whether PM exposure exacerbates acne-like inflammation and elucidate the underlying mechanisms. To confirm whether PM increases the messenger ribonucleic acid (mRNA) and protein levels of proinflammatory cytokines (IL-1α, IL-1β, IL-6, IL-8, and TNF-α) and cyclooxygenase (COX)-2 expression in *C*. *acnes-* or PGN-treated HEKn cells, we used quantitative real-time polymerase chain reactions, enzyme-linked immunosorbent assays, and western blot assays. The results demonstrated that *C*. *acnes*, PGN, and PM induced the expression of proinflammatory cytokines in a time- and dose-dependent manner at the mRNA and protein levels, respectively. Moreover, PM further increased the expression of proinflammatory cytokines, COX2, TLR4, and the phosphorylation of NF-κB in *C*. *acnes-* and PGN-treated HEKn cells. In conclusion, our results suggest that PM may exacerbate acne symptoms by increasing the inflammatory response.

## Introduction

Particulate matter (PM) refers to a heterogenous mixture of particles and droplets in the ambient atmosphere. These may include organic and inorganic particles, such as tobacco smoke, metals, dust, and pollen [1, 2]. Particles of 10 μm (PM_10_) or less (PM_2.5_), when inhaled into the lungs, can cause detrimental health effects. In particular, PM is significantly associated with cardiovascular and respiratory diseases [3–5]. There is growing evidence that air pollution is significantly associated with skin aging and inflammatory skin diseases such as atopic dermatitis, acne, and psoriasis [6–10]. The mechanisms by which PM may affect human skin involve skin barrier disruption and oxidative stress by reactive oxygen species, leading to the activation of inflammatory cascades [11].

Acne is a common inflammatory disease involving pilosebaceous units and results from increased sebum production, follicular keratinization, inflammation, and colonization by *Cutibacterium acnes* (*C*. *acnes*) [12, 13]. Although there is a lack of data, the detrimental effects of air pollutants on the skin and the worsening of acne severity have been recognized by most skin experts. Several studies have investigated the effects of pollution on acne vulgaris; however, it has not been proven whether PM aggravates acne inflammation [7, 8, 14]. Therefore, we aimed to investigate whether PM exposure exacerbates acne-like inflammation and elucidate the underlying mechanisms.

## Materials and methods

### Chemicals and preparation

The standard reference materials (SRM) 1649b were purchased from the National Institute of Standards and Technology (Gaithersburg, MD, USA) and dispersed in phosphate-buffered saline (PBS). *C*. *acnes* (KCTC 3314) was obtained from the Korean Collection for Type Cultures (KCTC, Daejeon, Korea), and peptidoglycan (PGN) from a *Staphylococcus aureus* cell wall component was purchased from Sigma-Aldrich (MO, USA). Specific antibodies against TLR4 and glyceraldehyde 3-phosphate dehydrogenase (GAPDH) were purchased from Santa Cruz Biotechnology (Santa Cruz, CA, USA). Antibodies against phospho-NF-κB p65, NF-κB p65, and cyclooxygenase (COX)-2 were purchased from Cell Signaling Technology (Danvers, MA, USA) and Abcam (Cambridge, UK). Human interleukin (IL)-6, IL-8, and tumor necrosis factor (TNF)-α enzyme-linked immunosorbent assay (ELISA) development kits were purchased from R&D Systems (Minneapolis, MN, USA).

### Cell culture

Primary neonatal human epidermal keratinocytes (HEKn, Cascade, Invitrogen, Carlsbad, CA, USA) were cultured in EpiLife medium (Cascade Biologics, Portland, OR, USA) supplemented with human keratinocyte growth supplement (HKGS, Cascade Biologics) at 37°C in a humidified atmosphere with 5% CO_2_. When the cultures reached 80% confluence, cells were dissociated into single cells using TrypLE Select Enzyme (Gibco, Waltham, USA) for 5 min at room temperature. The HEKn cells used in the experiments were between passages three and five.

### Bacterial culture

Reinforced clostridial liquid and solid medium (RCM, Difco Laboratories, Detroit, MI, USA) were used to grow *C*. *acnes* for 48–72 h at 37°C under anaerobic conditions (5% H_2_, 5% CO_2_, and 90% N_2_). *C*. *acnes* suspensions were centrifuged at 4,000 rpm for 10 min at 4°C and then washed three times with cold PBS. Finally, the cell number was estimated by measuring the optical density of the suspension at 600 nm using a spectrophotometer. As we previously observed that an OD_600_ = 1.0 is equivalent to 5.0 × 10^8^ colony forming units (CFU) per 1 mL, the number of bacterial cells was adjusted with PBS to 5×10^8^ CFU/mL. To obtain heat-killed bacteria, a *C*. *acnes* suspension (5 × 10^8^ CFU/mL) was heated at 80°C for 30 min. Heat-killed *C*. *acnes* were maintained at 4°C and centrifuged at 13,200 rpm for 10 min before use.

### Cell viability assay

Cell viability was assessed using a modified 3-(4,5-dimethylthiazol-2-yl)-2,5-diphenyl-2H-tetrazolium bromide (MTT, M5655, Sigma) assay. The cells were seeded at 70% confluence in each well of a 96-well plate. After 24 h, the cells were treated with several concentrations of heat-killed *C*. *acnes* (50, 100, 500, and 1000 Multiplicity of infection (MOI)) and PM (5, 10, 25, and 50 μg/cm^2^). After incubation for 3, 6, 12, and 24 h, 10 μL of MTT solution (5 mg/mL in PBS) was added to each well. Cells were incubated for a further 3 h at 37°C. Supernatants were removed and 100 μL of dimethyl sulfoxide (DMSO) solution was added to each well. Finally, spectrophotometric absorbance was measured at 570 nm.

### Ribonucleic acid (RNA) isolation and real-time quantitative polymerase chain reaction (qRT-PCR)

Total ribonucleic acid was extracted from HEKn cells using the TRIzol reagent (Invitrogen) according to the manufacturer’s instructions. Next, cDNA was synthesized from total RNA using the RevertAid First Strand cDNA Synthesis Kit (Thermo Scientific, Waltham, USA). Moreover, qRT-PCR assays were performed with a real-time thermal cycler (Applied Biosystems, Foster City, CA, USA) using PowerUp SYBR Green Master Mix (Applied Biosystems). The PCR data were normalized according to GAPDH levels, and relative quantitation was performed using the comparative 2-ΔΔCt method.

### Western blot analysis

Cell lysates were centrifuged, and protein concentration was determined using the bicinchoninic acid assay (BCA) method. Equal concentrations of protein were loaded for 8–10% sodium dodecyl sulfate-polyacrylamide gel electrophoresis and transferred to nitrocellulose membranes. After blocking with 5% skimmed milk for 1 h, the membrane was incubated with primary antibodies (24 h) and anti-rabbit horseradish peroxidase-conjugated antibodies (1 h). The protein expression of NF-κB, TLR2, and COX2 was detected using the EzWestLumi plus system (ATTO, Tokyo, Japan) and ChemiDoc™ XRS image analyzer (Bio-Rad, Hercules, CA, USA).

### ELISA

After exposure to heat-killed *C*. *acnes* or PGN and PM 1649b for 24 h, the supernatants were collected and stored at -70°C until assays were performed. The protein expression levels of IL-6, IL-8, and TNF-α in HEKn cells were measured using specific ELISA kits, according to the manufacturer’s recommended protocols.

### Statistical analysis

We compared the resulting PM effects in the treatment groups and controls using one-way analysis of variance and Tukey’s multiple-comparison post hoc test. Differences between groups were considered significant at *P* < 0.05, and statistical analyses were performed using GraphPad Prism 8.0 (GraphPad Software, Inc., CA, USA).

## Results

### Effect of PM and *C*. *acnes* on the viability of HEKn cells

**Fig 1:**
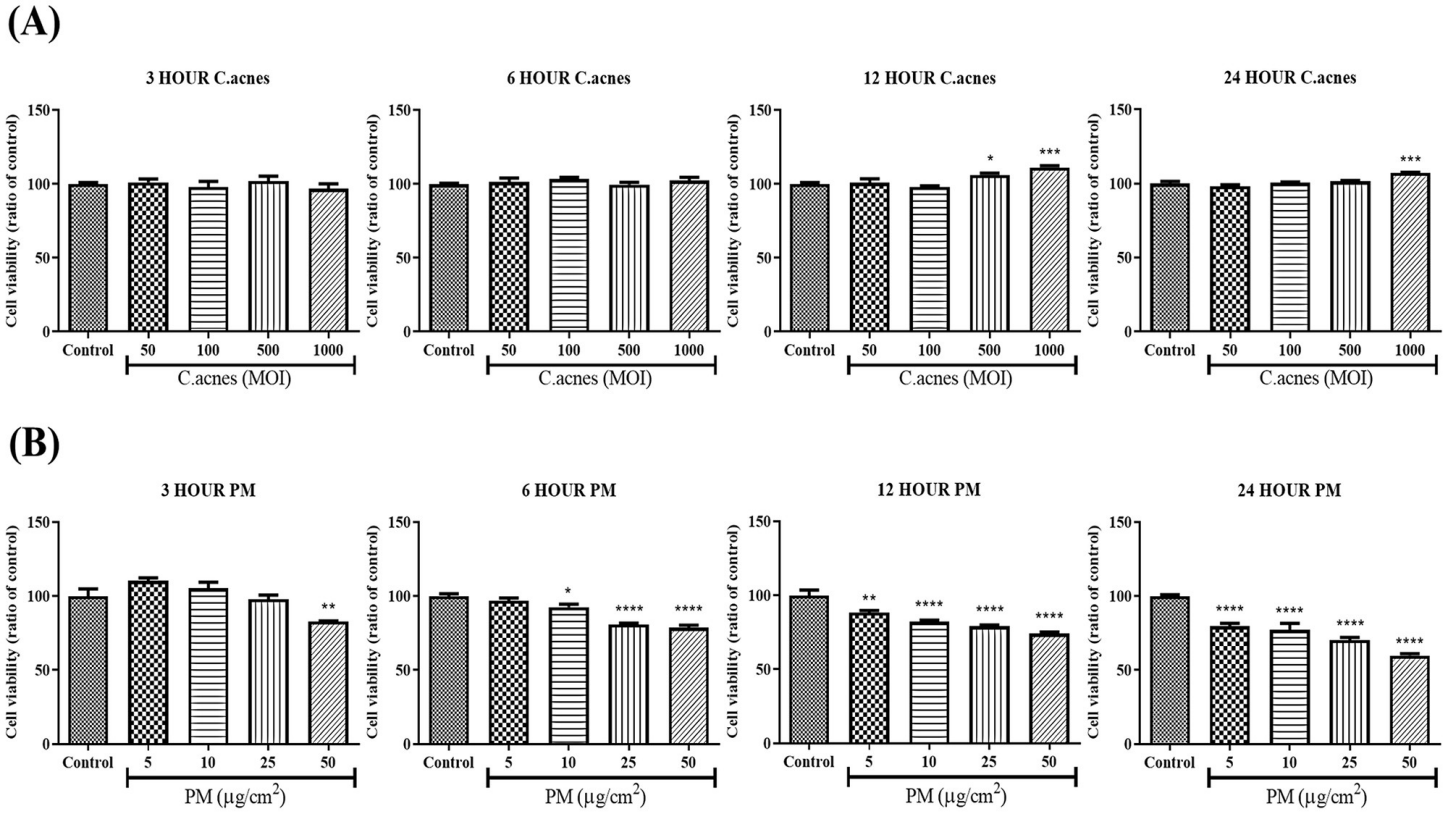
Effect of PM and *C*. *acnes* on the viability of HEKn cells. Cytotoxicity of heat-killed *C*. *acnes* and PM on HEKn cells determined by the MTT assay. (A) HEKn cells were treated with several concentrations of (A) heat-killed *C*. *acnes* (50–1000 MOI) and (B) PM (5–50 μg/cm^2^) for 3–24 h. Data are shown as mean + standard error of the mean (SEM). Statistical significance was determined by repeated measures ANOVA, post hoc Bonferroni correction, comparing to control (*p < 0.05, **p < 0.01, ***p<0.001, ****p<0.0001).

To choose a suitable concentration and time point for HEKn cell treatment, we investigated the cytotoxicity of several concentrations of *C*. *acnes* (50, 100, 500, and 1000 MOI) and PM (5, 10, 25, and 50 μg/cm^2^) and time points of 3, 6, 12, and 24 h. The data from the MTT assay showed no significant change in the viability of HEKn cells treated with *C*. *acnes* (Fig 1A). In contrast, significant cytotoxicity was observed after PM treatment in a dose- and time-dependent manner (Fig 1B). As PM-treated cells exhibited < 70% cell viability at concentrations above 25 μg/cm^2^ for 24 h, the cells were treated with PM at a concentration of 10 μg/cm^2^ in further experiments.

### Proinflammatory cytokine expression induced by *C*. *acnes*, PGN, and PM

Proinflammatory cytokines such as IL-1β, IL-6, IL-8, and TNF-α are responsible for the follicular keratinization and inflammation of acne [15]. Quantitative RT-PCR and ELISA analyses were performed to investigate the effect of *C*. *acnes*, PGN, or PM on the expression of proinflammatory cytokines. HEKn cells were treated with several concentrations of heat-killed *C*. *acnes* (100, 500, 1000 MOI), PGN (1, 10, and 25 μg/mL), and PM (5, 10, 25 μg/cm^2^). The relative mRNA levels of IL-1α, IL-1β, IL-6, IL-8, and TNF-α increased in a dose-dependent manner after treatment with *C*. *acnes*, PGN, and PM (S1 Fig). The results of the ELISA assay also confirmed that treatment with *C*. *acnes*, PGN, and PM significantly increased the expression of IL-6, IL-8, and TNF-α (S2 Fig). Since IL-8 has a role in the pathogenesis of acne [16], we selected the concentration of *C*. *acnes* of 500 MOI for further experiments based on our ELISA data.

### Effect of PM on proinflammatory cytokine expression in *C*. *acnes* (500 MOI) or PGN (10 μg/mL) treated HEKn cells

**Fig 2:**
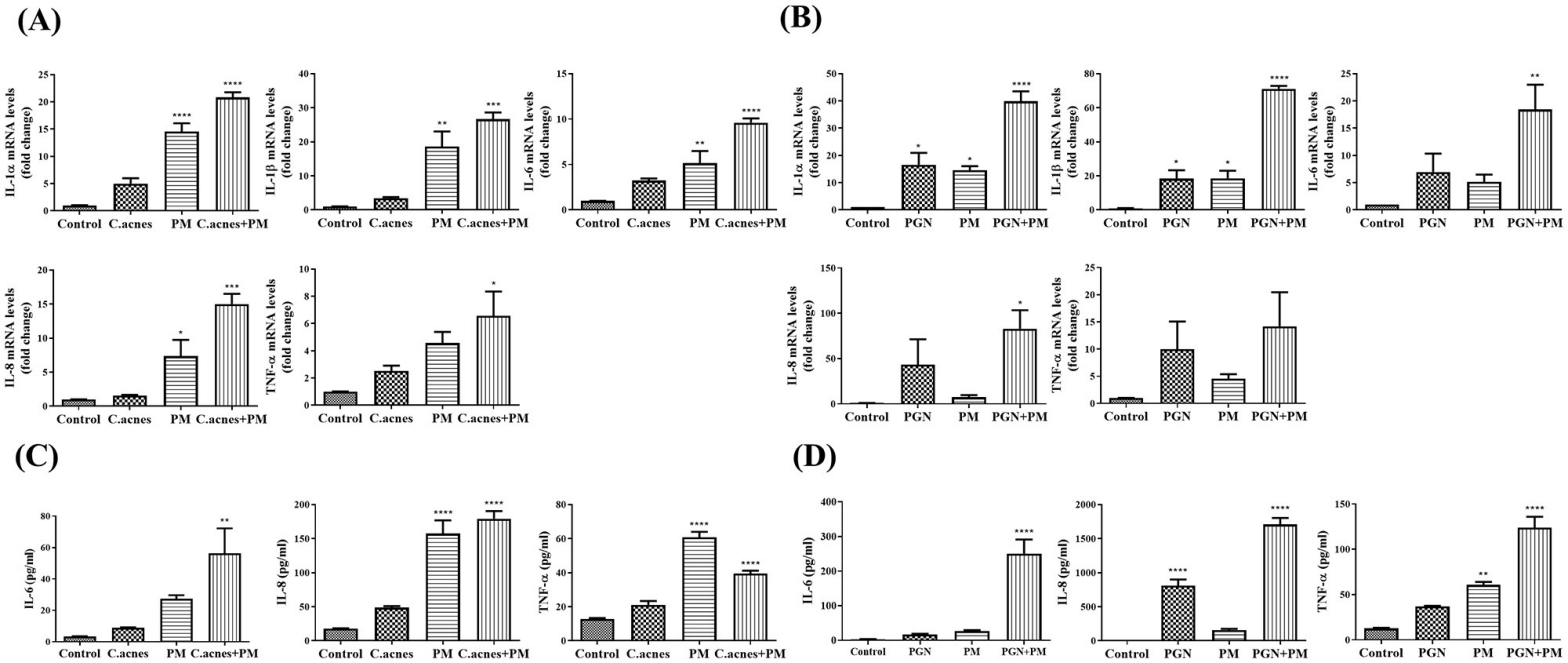
PM increases the expression of proinflammatory cytokines in *C*. *acnes* (500 MOI), or PGN (10 μg/mL) treated HEKn cells. HEKn cells are co-treated with PM (10 μg/cm^2^) and heat-killed *C*. *acnes* (500 MOI) or PGN (10 μg/mL) for 24 h. qRT-PCR results demonstrate that PM increases the mRNA expression of IL-1α, IL-1β, IL-6, IL-8, and TNF-α in (A) *C*. *acnes* and (B) PGN-treated cells. The relative mRNA levels are presented as fold change compared with untreated cells (0 μg/cm^2^). ELISA results show that PM also increases the protein expression of proinflammatory cytokines in (C) *C*. *acnes* or (D) PGN-treated cells. Data are shown as mean + SEM. Statistical significance was determined by repeated measures ANOVA, post hoc Bonferroni correction, comparing to control (*p < 0.05, **p < 0.01, ***p<0.001, ****p<0.0001).

We examined whether PM could upregulate the expression of proinflammatory cytokines (IL-1α, IL-1β, IL-6, IL-8, and TNF-α) in *C*. *acnes-* or PGN-treated HEKn cells. HEKn cells were seeded in six-well plates, allowed to proliferate until 70% confluence, and subsequently subjected to HKGS starvation for 4 h in EpiLife medium. HEKn cells were co-treated with PM (10 μg/cm^2^) and heat-killed *C*. *acnes* (500 MOI) or PGN (10 μg/mL) at 37°C. After 3 h (mRNA) and 24 h (protein), the supernatants were collected, and the mRNA and protein expression levels of proinflammatory cytokines were determined using qRT-PCR and ELISA. The data revealed that PM further increased the expression of proinflammatory cytokines, such as IL-1α, IL-1β, IL-6, IL-8, and TNF-α, in HEKn cells treated with *C*. *acnes* or PGN (Fig 2). This suggests that PM amplifies *C*. *acnes-* and PGN-induced inflammation.

### Effect of PM on the COX2 expression in *C*. *acnes* (500 MOI) or PGN (10 μg/mL) treated HEKn cells

**Fig 3:**
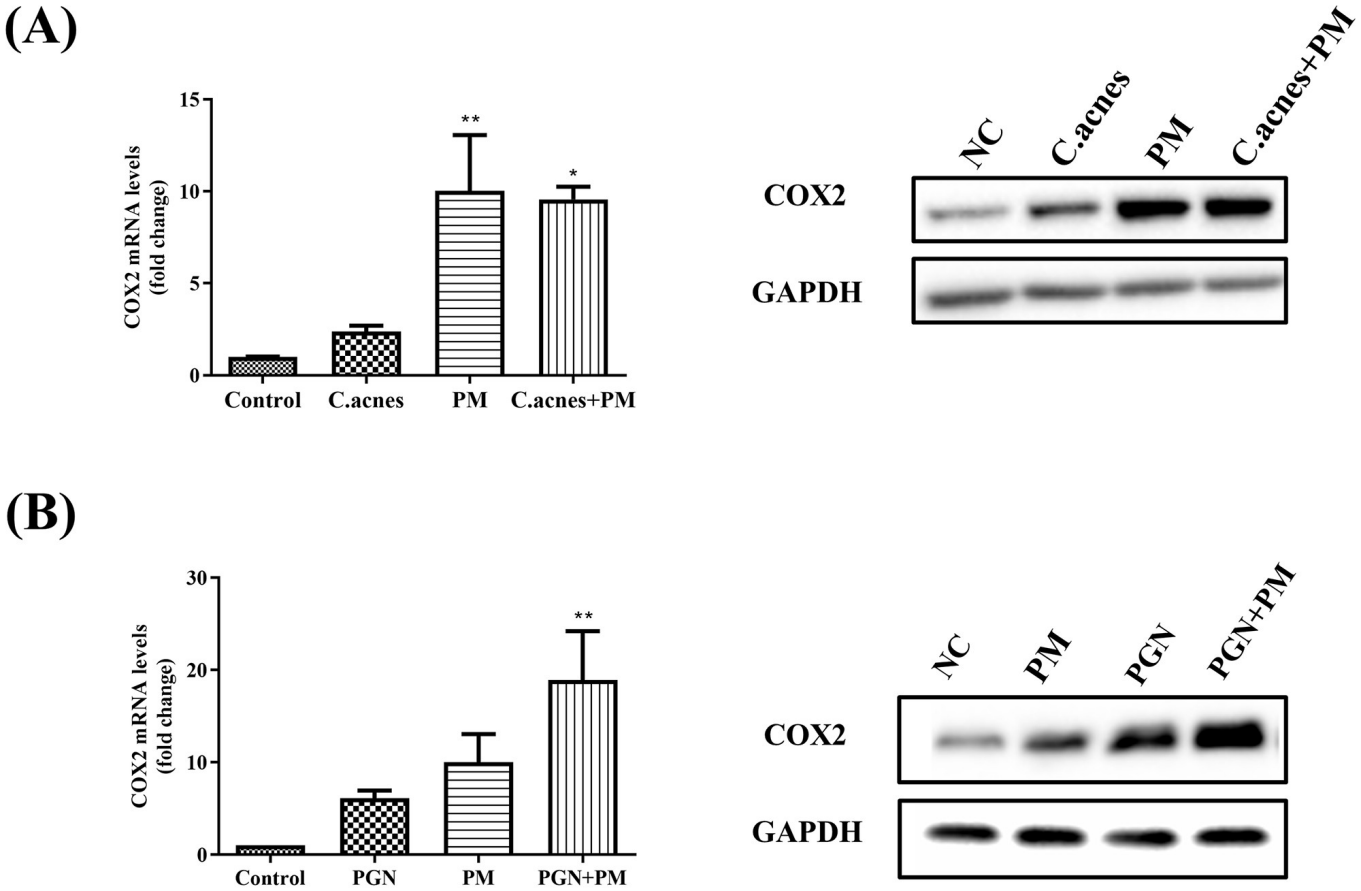
PM enhances COX2 expression induced by *C*. *acnes* (500 MOI) or PGN (10 μg/mL) in HEKn cells. PM (10 μg/cm^2^) increases the level of COX2 expression in HEKn cells treated with *C*. *acnes* (A) or PGN (B). The mRNA and protein expression levels of COX2 were assessed using qRT-PCR and western blotting. Data are shown as mean + SEM. P-value determined by repeated measures ANOVA, post hoc Bonferroni correction, comparing to control (*p < 0.05, **p < 0.01).

COX2 is an enzyme associated with inflammation and control of cell growth [17, 18]. In particular, several studies have demonstrated that COX2 expression is selectively upregulated in inflammatory acne lesions [19]. Therefore, we investigated whether COX2 expression in HEKn cells co-treated with *C*. *acnes* and PM increased compared to cells treated with *C*. *acnes* alone. Our results showed that *C*. *acnes* and PGN induced COX2 expression in HEKn cells. In addition, PM treatment significantly increased the mRNA and protein expression levels of COX2 (Fig 3).

### PM intensified the inflammation induced by *C*. *acnes* and PGN through activation of the NF-κB signaling pathway

**Fig 4:**
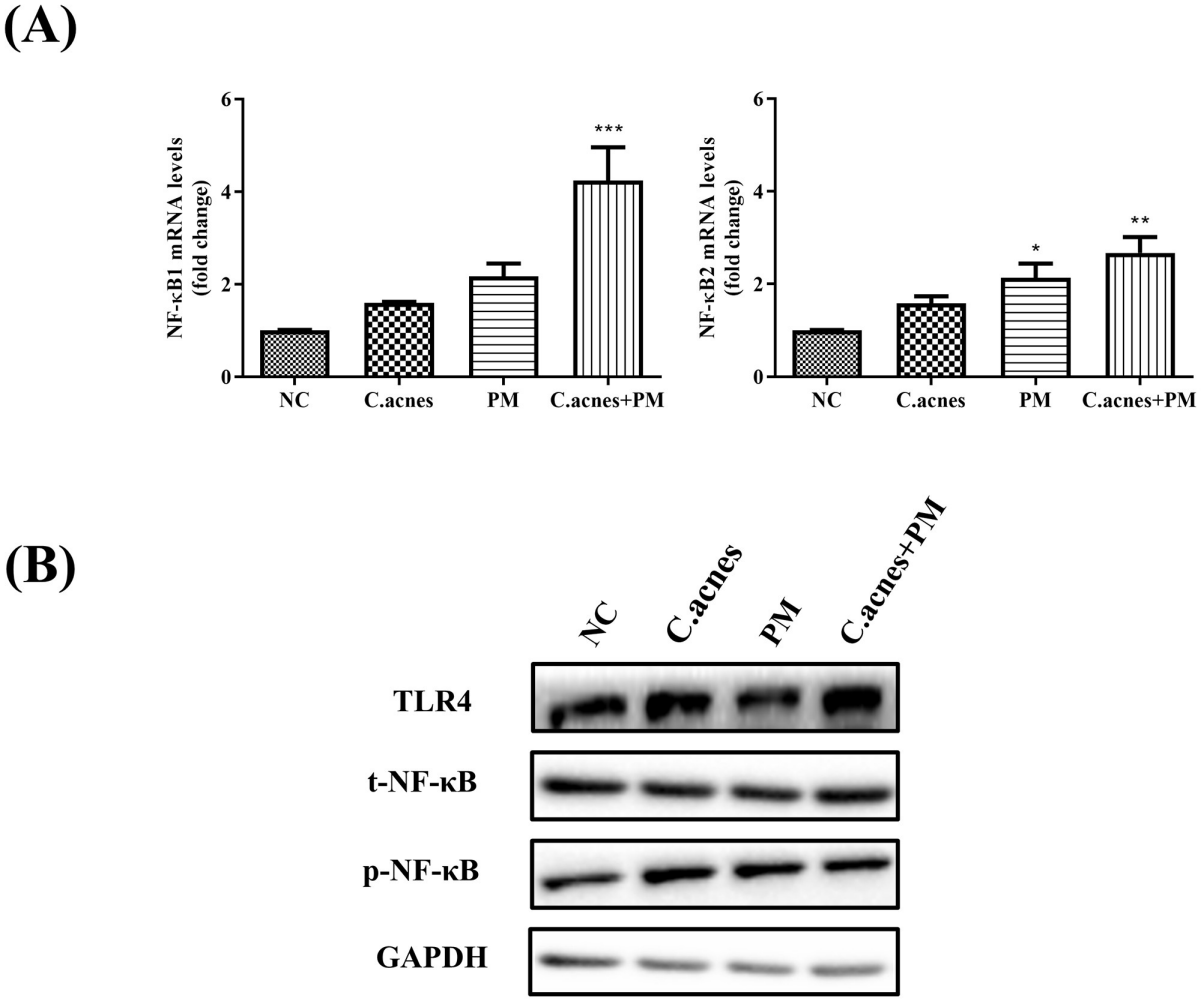
PM significantly induces the activation of the TLR/NF-κB signaling pathway in *C*. *acnes*-treated HEKn cells. PM increases the expression of TLR4 and NF-κB in HEKn cells co-treated with PM (10 μg/cm^2^) and heat-killed *C*. *acnes* (500 MOI). (A) The mRNA expressions of NF-κB1 and NF-κB2 were analyzed by qRT-PCR. (B) Western blot analysis was employed to measure the protein expression levels of NF-κB and TLR4. Data are shown as mean + SEM. P-value determined by repeated measures ANOVA, post hoc Bonferroni correction, compared with control (*p < 0.05, **p < 0.01, ***p<0.001). p-NF-kB, phospho-NF-κB; t-NF-kB, total-NF-kB.

The nuclear factor kappa-B (NF-κB) pathway, which is activated in response to various stimuli, has been considered a key transcriptional modulator of inflammatory processes. After activation, NF-κB penetrates the nucleus and upregulates COX2 gene expression [20]. Recent studies have reported that *C*. *acnes* may trigger inflammation through activation of toll-like receptors (TLR2 and TLR4) in keratinocytes, leading to the activation of specific signaling cascades, including the NF-κB and mitogen-activated protein kinase pathways, resulting in the induction of immune response genes [21]. Thus, to elucidate the possible effects of PM on *C*. *acnes*-induced inflammatory signaling pathways, we evaluated the effects of PM on the expression of TLR/NF-κB pathway signaling molecules. The phosphorylation of NF-κB and TLR4 was increased by *C*. *acnes* and PGN. As shown in Fig 4, PM upregulated the phosphorylation of NF-κB1, NF-κB2, and TLR4 expression in HEKn cells. These data suggest that PM amplifies the inflammatory reaction by increasing the activity of phospho-NF-κB in HEKn cells induced by *C*. *acnes* or PGN.

## Discussion

Inflammation has been suggested as a key factor involved in the development and aggravation of acne vulgaris [22]. For example, IL-1 can trigger remodeling of the pilosebaceous units and promote comedogenesis by follicular hyperkeratinization. In addition, prostaglandin E2, which is mediated by COX2, has been shown to cause sebaceous gland hyperplasia and sebum overproduction [22]. Several studies have pointed out that PM can negatively affect the skin barrier by inducing inflammation and oxidative stress [8, 23]. Since little is known about the effects of PM on acne, we aimed to investigate whether PM aggravates acne inflammation by assessing the proinflammatory cytokine expression in human epidermal keratinocytes. Previous reports demonstrated that PGN from *S*. *aureus* can activate the NF-κB pathway and induce inflammatory cytokine production via TLR activation [24–26]. Thus, we used PGN to mimic *C*. *acnes*-induced cellular responsiveness *in vitro* and observe expression of inflammatory cytokines.

Our results showed that PM increased the production of proinflammatory cytokines (IL-1α, IL-1β, IL-6, IL-8, and TNF-α), and of COX2, and promoted the phosphorylation of NF-κB induced by *C*. *acnes* in HEKn cells. The group treated with both *C*. *acnes* and PM showed increased expression of phospho-NF-κB and TLR4 compared to the groups treated only with *C*. *acnes* or PGN. Taken together, these results demonstrate that PM has proinflammatory properties, with an emphasis on the activation of the TLR/NF-κB pathway, thus aggravating the *C*. *acnes*-induced inflammatory response.

It has been proposed that TLR2 may play a pivotal role in the induction of *C*. *acnes*-induced inflammation, including cytokine production [27, 28]. The expression of IL-6 and TNF-α is significantly inhibited by a TLR2 inhibitor in human keratinocytes or TLR2-deficient murine keratinocytes [29]. In addition, TLR2 and TLR4 have been reported to be involved in the inflammation caused by *C*. *acnes*. Nagy et al. showed that *C*. *acnes* increased the hBD2 and IL-8 mRNA levels in keratinocytes and that *C*. *acnes*-induced upregulation in gene expression is dependent on both TLR2 and TLR4 [30]. In our study, *C*. *acnes* significantly induced the expression of TLR4 in keratinocytes, which was further increased by PM. Our findings reveal a potent role of TLR4 in *C*. *acnes*-induced inflammation and suggest that *C*. *acnes* mediates different regulatory factors in response to different TLR signaling pathways in keratinocytes.

However, this study has several limitations. While it includes several key factors in the pathogenesis of acne, further *in vitro* research using sebocytes is required, and additional factors are needed to mimic the *in vivo* conditions of acne. Although *C*. *acnes* is the most well-known pathogen involved in the pathogenesis of acne, recent studies suggest that other bacteria can directly and indirectly contribute to the inflammatory process in acne. For example, *Staphylococcus epidermidis* produce virulence factors that inhibit the growth of *C*. *acnes*, and *Cutibacterium granulosum*, which is highly abundant in acne lesions, also generates virulence factors [29, 31]. Moreover, it has been shown that *Malassezia restricta* and *Malassezia globosa* are abundant in young patients with acne [32, 33]. Therefore, the presence of other microorganisms and their interactions should also be considered.

To the best of our knowledge, no previous reports have been published on the direct effect of PM on acne inflammation. Our results suggest that PM may potentially aggravate acne by amplifying the inflammatory response via upregulation of the TLR/NF-κB pathway. Based on our findings, the inhibition of TLR/NF-κB signaling may be a promising target for the prevention and treatment of PM-induced acne exacerbation. Further studies with TLR/NF-κB pathway inhibitors and acne microenvironment models are needed to investigate the *in vivo* effects of PM on acne inflammation and to elucidate the underlying mechanisms.

## Supporting information

S1 Fig mRNA expression levels of proinflammatory cytokines in *C*. *acnes*, PGN, or PM treated HEKn cells. HEKn cells are treated with various concentrations of (A) heat-killed *C*. *acnes* (100–1000 MOI), (B) PGN (1–25 μg/mL), and (C) PM (5–25 μg/cm^2^) for 3 h. The mRNA expression levels of IL-1α, IL-1β, IL-6, and TNF-α are determined using qRT-PCR. Data are shown as mean + SEM. Statistical significance was determined by repeated measures ANOVA, post hoc Bonferroni correction, comparing to control (*p < 0.05, **p < 0.01, ***p<0.001, ****p<0.0001).
(TIF)

S2 Fig Protein expression levels of proinflammatory cytokines in *C*. *acnes*, PGN, or PM treated HEKn cells. HEKn cells are treated with various concentrations of (A) heat-killed *C*. *acnes* (100–1000 MOI), (B) PGN (1–25 μg/mL), and (C) PM (5–25 μg/cm^2^) for 24 h. The protein expression levels of IL-1, IL-6, and TNF-α are determined using western blot analysis. Data are shown as mean + SEM. P-value determined by repeated measures ANOVA, post hoc Bonferroni correction, comparing to control (*p < 0.05, **p < 0.01, ***p<0.001, ****p<0.0001).
(TIF)

S3 Fig (PNG)

S4 Fig (PNG)

S5 Fig (PDF)
