# Supplementary figures and images for: Particulate matter increases Cutibacterium acnes-induced inflammation in human epidermal keratinocytes via the TLR4/NF-κB pathway
Source: PLoS One. 2022 Aug 10;17(8):e0268595. doi: 10.1371/journal.pone.0268595 (PMC9365135; doi:10.1371/journal.pone.0268595)

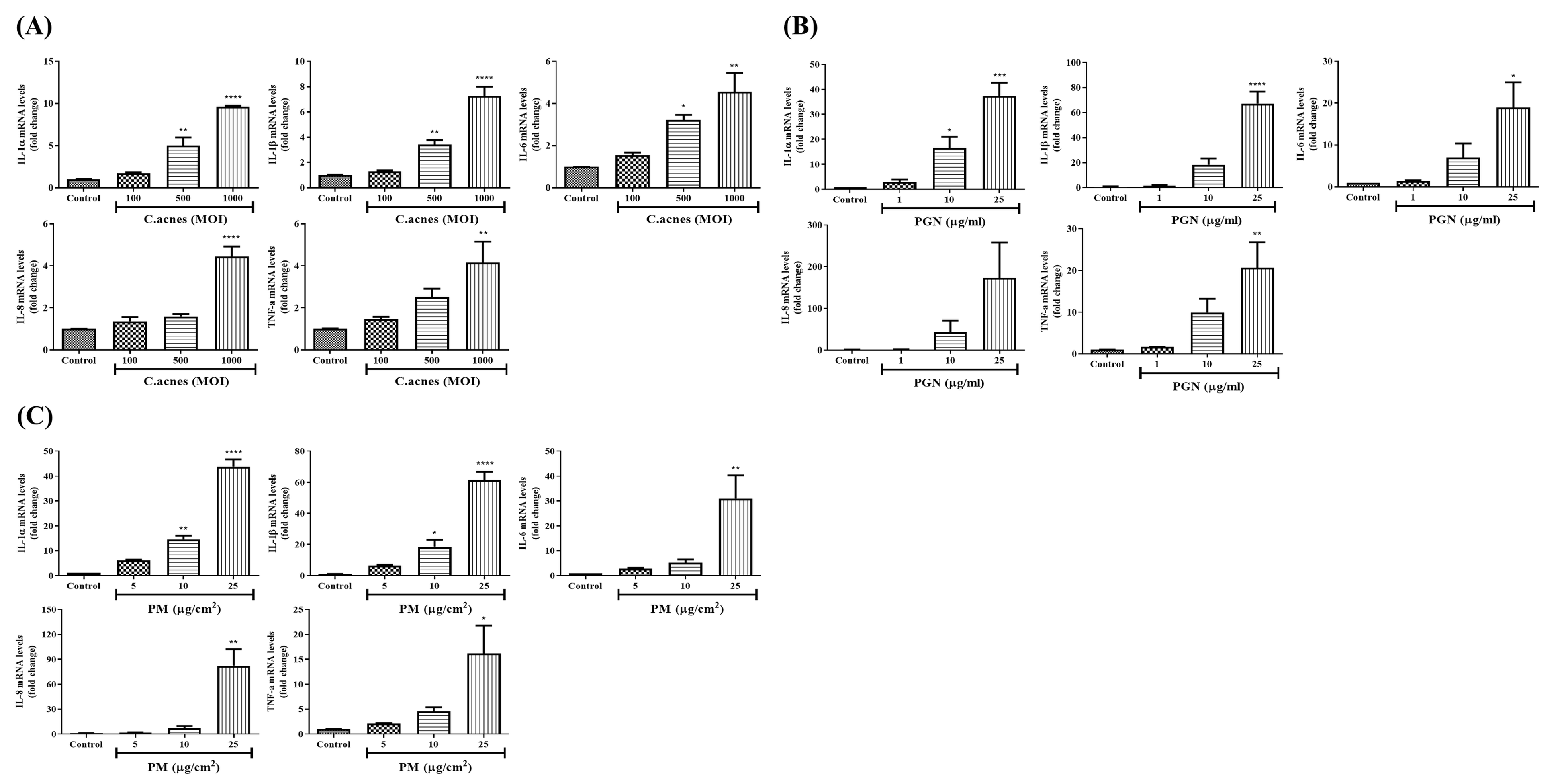

Supplement: S1 Fig — HEKn cells are treated with various concentrations of (A) heat-killed C. acnes (100–1000 MOI), (B) PGN (1–25 μg/mL), and (C) PM (5–25 μg/cm2) for 3 h. The mRNA expression levels of IL-1α, IL-1β, IL-6, and TNF-α are determined using qRT-PCR. Data are shown as mean + SEM. Statistical significance was determined by repeated measures ANOVA, post hoc Bonferroni correction, comparing to control (*p < 0.05, **p < 0.01, ***p<0.001, ****p<0.0001). (TIF) [file pone.0268595.s001.tif]

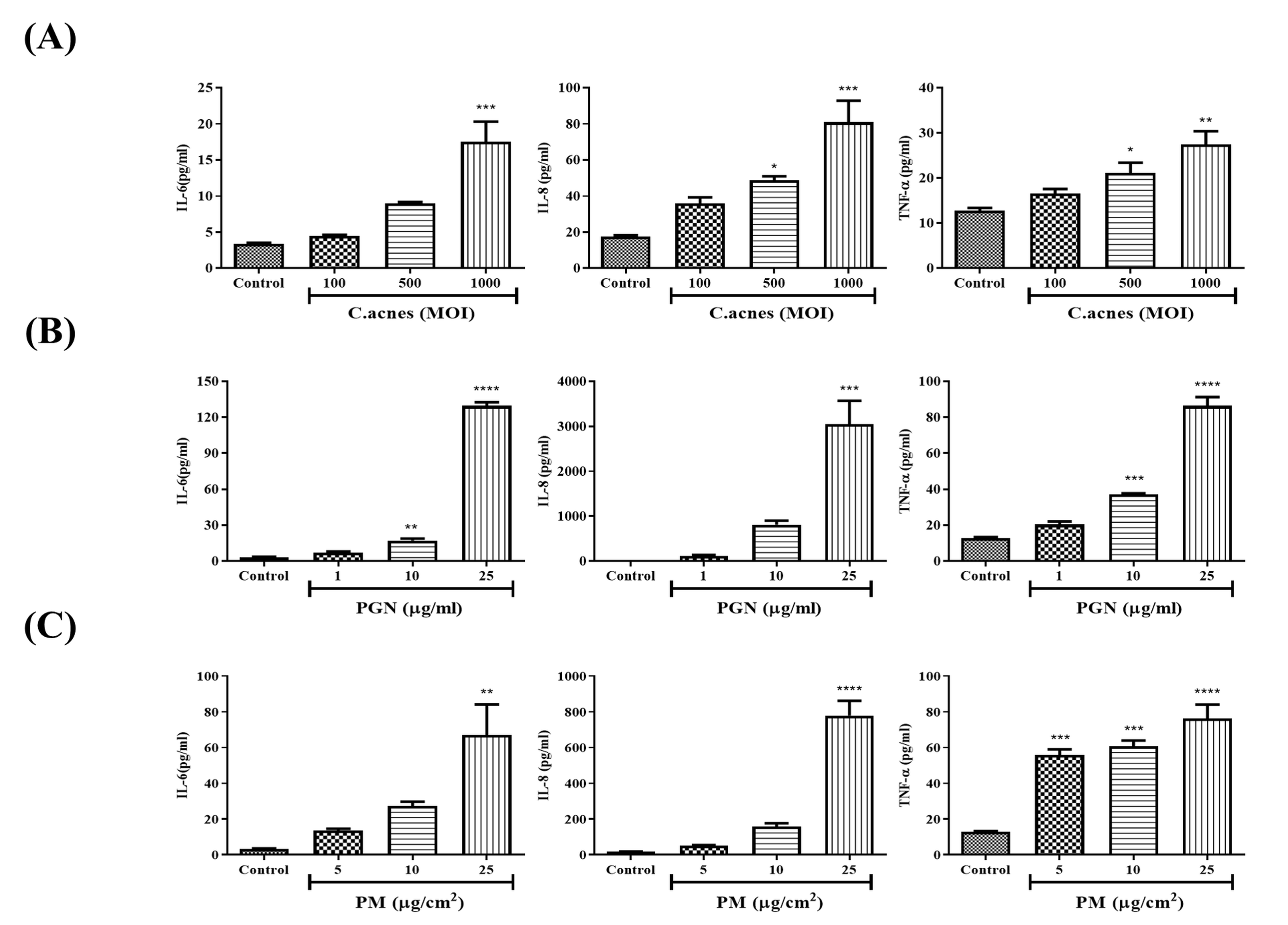

Supplement: S2 Fig — HEKn cells are treated with various concentrations of (A) heat-killed C. acnes (100–1000 MOI), (B) PGN (1–25 μg/mL), and (C) PM (5–25 μg/cm2) for 24 h. The protein expression levels of IL-1, IL-6, and TNF-α are determined using western blot analysis. Data are shown as mean + SEM. P-value determined by repeated measures ANOVA, post hoc Bonferroni correction, comparing to control (*p < 0.05, **p < 0.01, ***p<0.001, ****p<0.0001). (TIF) [file pone.0268595.s002.tif]

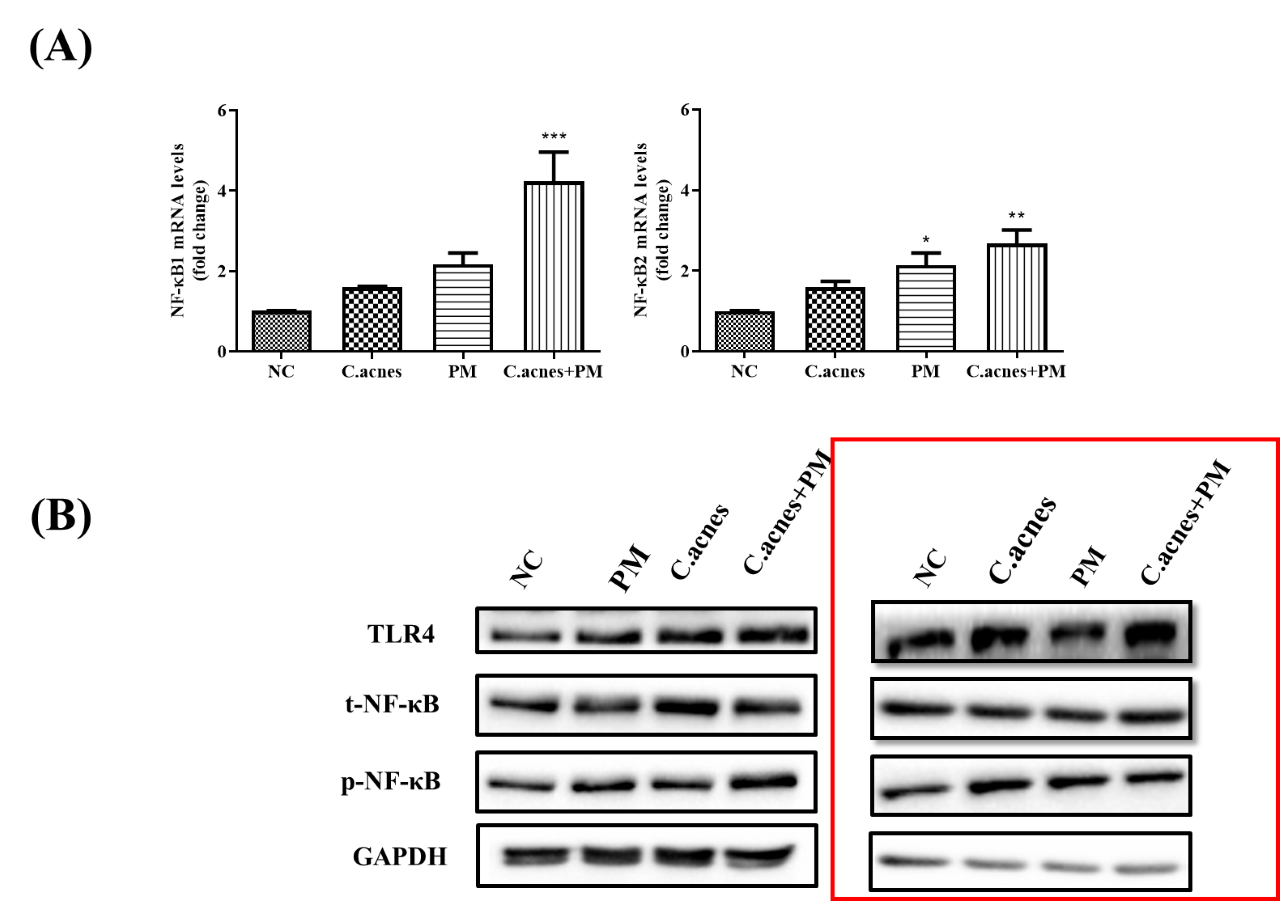

Supplement: S3 Fig — (PNG) [file pone.0268595.s003.png]

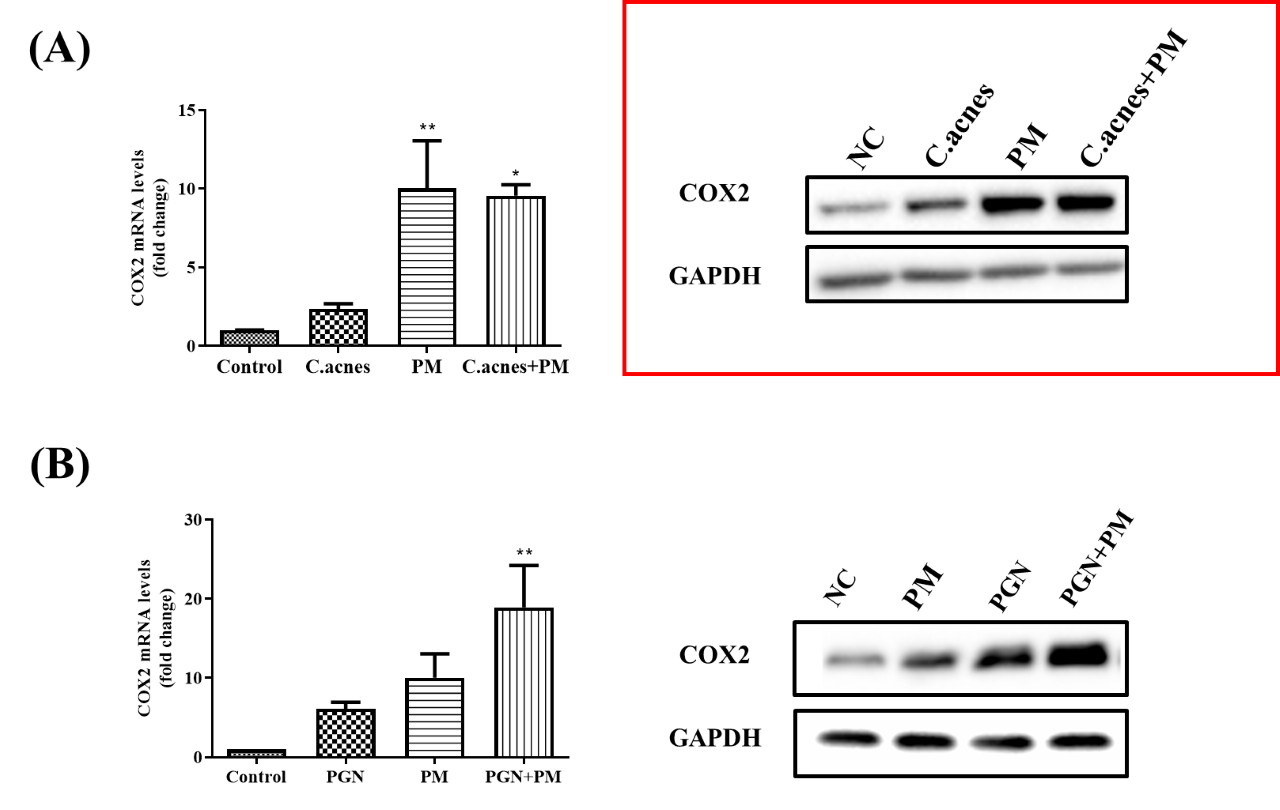

Supplement: S4 Fig — (PNG) [file pone.0268595.s004.png]

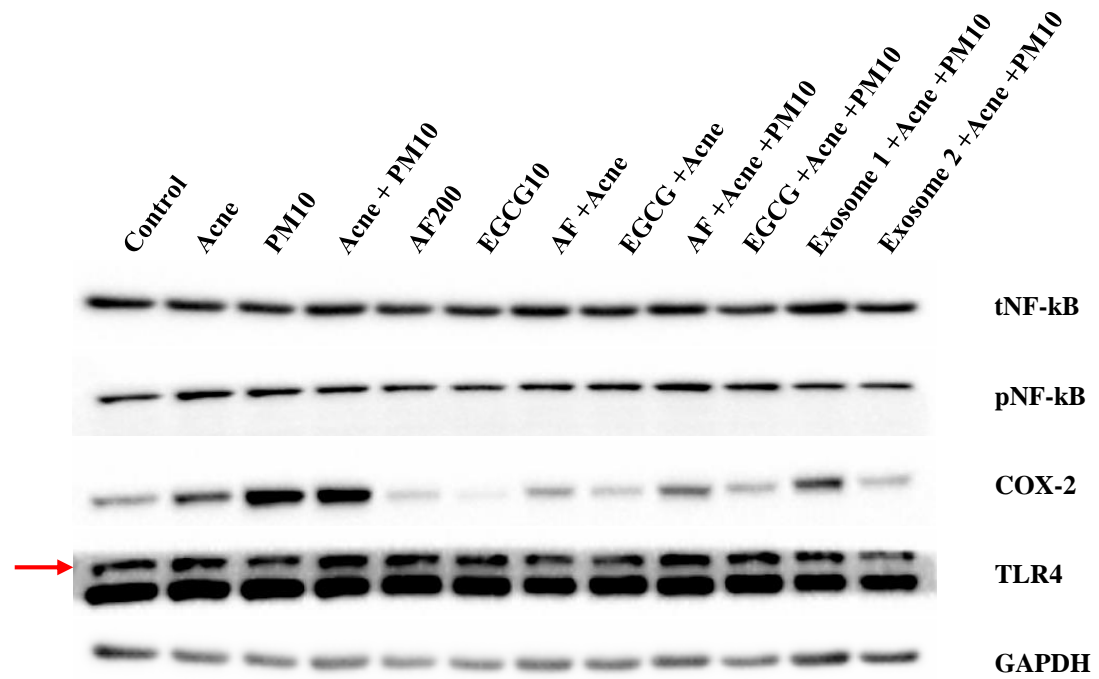

**tNF-kB**

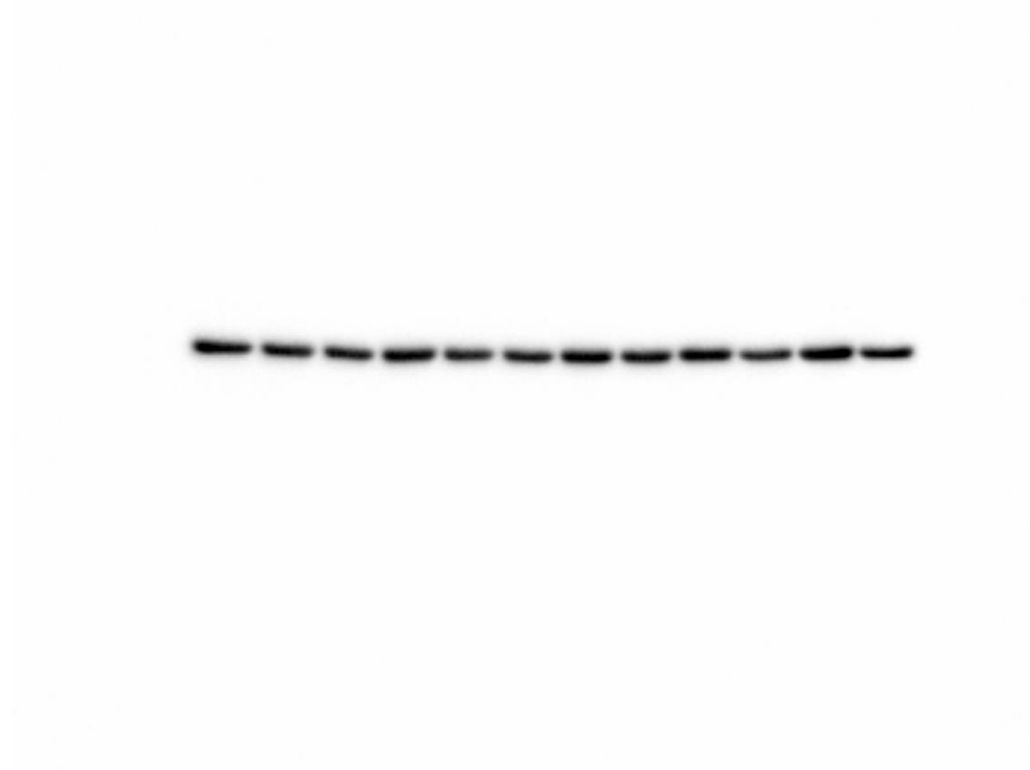

**pNF-kB**

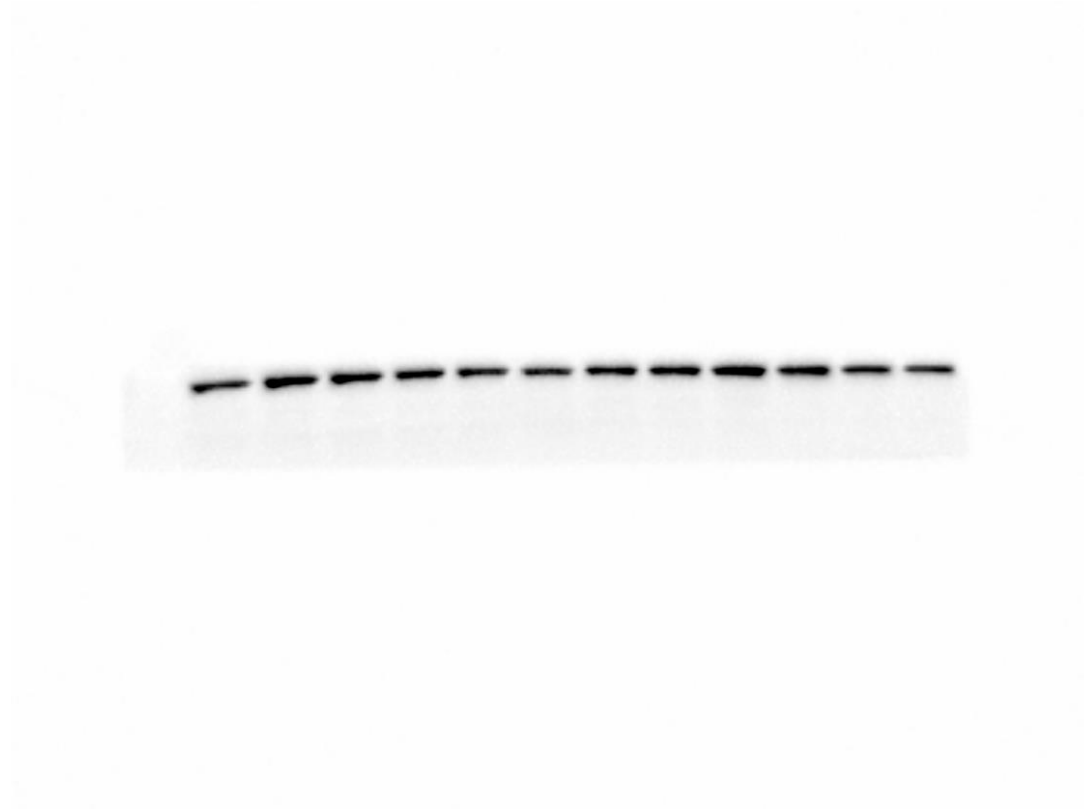

**COX-2**

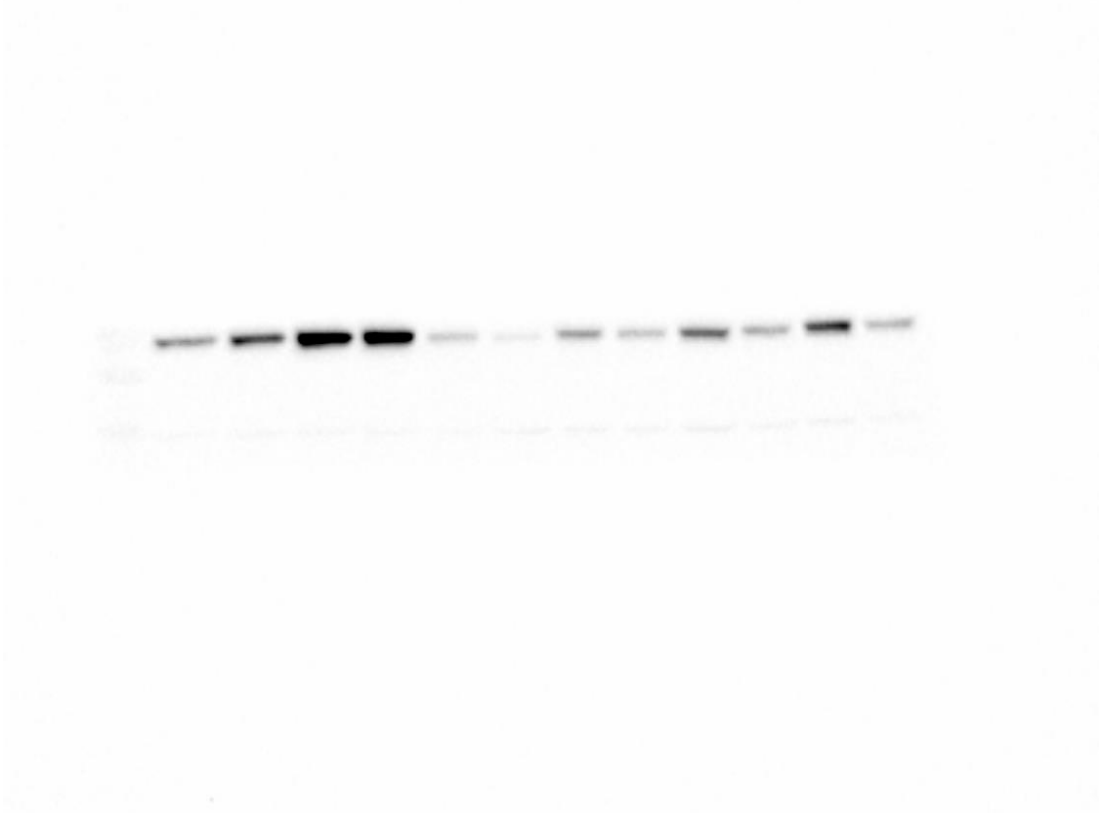

**TLR4**

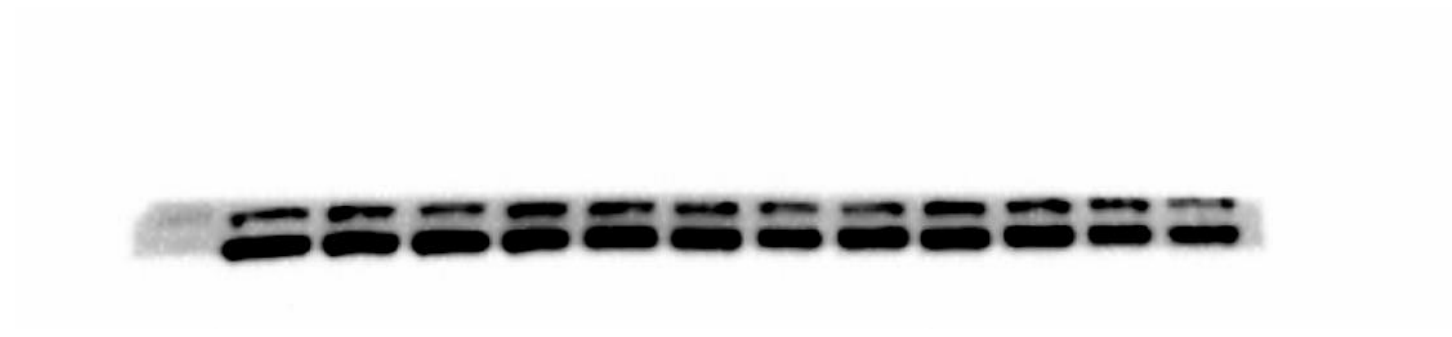

**GAPDH**

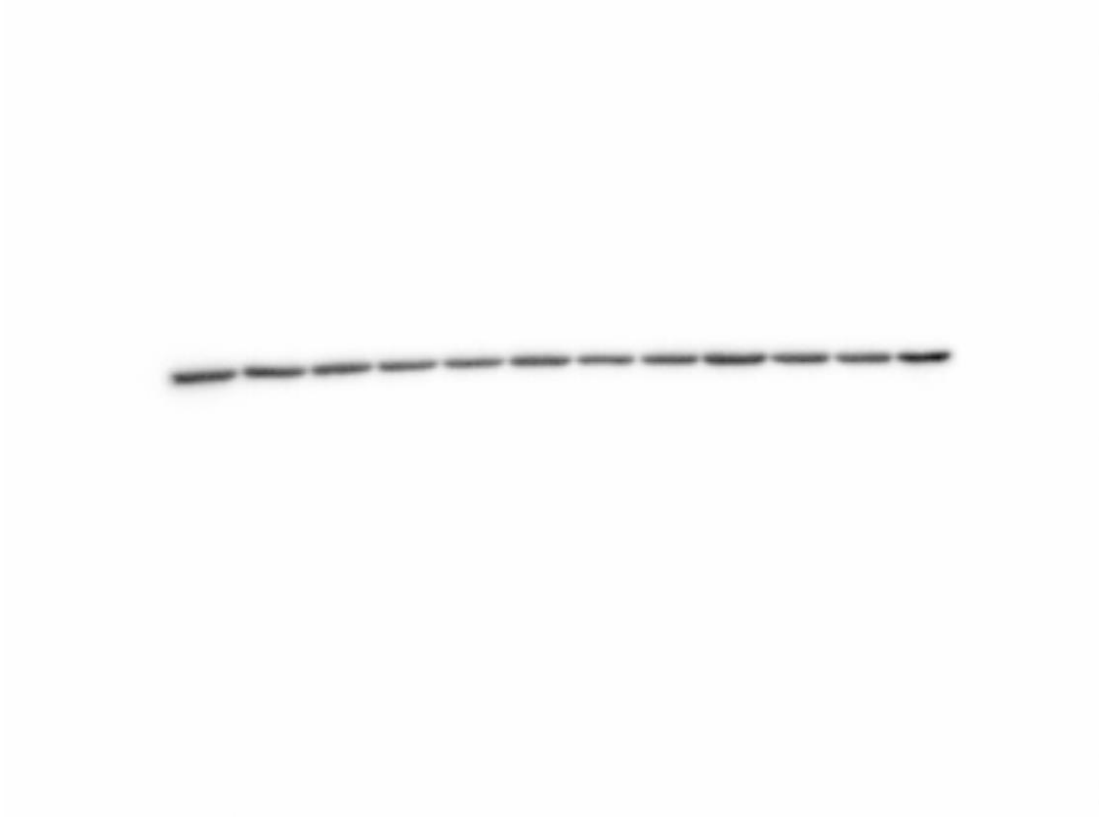

Supplement: S5 Fig — (PDF) [file pone.0268595.s005.pdf]
